# Integrated radiomic framework for breast cancer and tumor biology using advanced machine learning and multiparametric MRI

**DOI:** 10.1038/s41523-017-0045-3

**Published:** 2017-11-14

**Authors:** Vishwa S. Parekh, Michael A. Jacobs

**Affiliations:** 1 The Russell H. Morgan Department of Radiology and Radiological Science, Division of Cancer Imaging, The Johns Hopkins School of Medicine, Baltimore, MD 21205 USA; 2 Department of Computer Science, The Johns Hopkins University, Baltimore, MD 21208 USA; 3 Sidney Kimmel Comprehensive Cancer Center, The Johns Hopkins School of Medicine, Baltimore, MD 21205 USA

## Abstract

Radiomics deals with the high throughput extraction of quantitative textural information from radiological images that not visually perceivable by radiologists. However, the biological correlation between radiomic features and different tissues of interest has not been established. To that end, we present the radiomic feature mapping framework to generate radiomic MRI texture image representations called the radiomic feature maps (RFM) and correlate the RFMs with quantitative texture values, breast tissue biology using quantitative MRI and classify benign from malignant tumors. We tested our radiomic feature mapping framework on a retrospective cohort of 124 patients (26 benign and 98 malignant) who underwent multiparametric breast MR imaging at 3 T. The MRI parameters used were T1-weighted imaging, T2-weighted imaging, dynamic contrast enhanced MRI (DCE-MRI) and diffusion weighted imaging (DWI). The RFMs were computed by convolving MRI images with statistical filters based on first order statistics and gray level co-occurrence matrix features. Malignant lesions demonstrated significantly higher entropy on both post contrast DCE-MRI (Benign-DCE entropy: 5.72 ± 0.12, Malignant-DCE entropy: 6.29 ± 0.06, *p* = 0.0002) and apparent diffusion coefficient (ADC) maps as compared to benign lesions (Benign-ADC entropy: 5.65 ± 0.15, Malignant ADC entropy: 6.20 ± 0.07,* p* = 0.002). There was no significant difference between glandular tissue entropy values in the two groups. Furthermore, the RFMs from DCE-MRI and DWI demonstrated significantly different RFM curves for benign and malignant lesions indicating their correlation to tumor vascular and cellular heterogeneity respectively. There were significant differences in the quantitative MRI metrics of ADC and perfusion. The multiview IsoSVM model classified benign and malignant breast tumors with sensitivity and specificity of 93 and 85%, respectively, with an AUC of 0.91.

## Imaging: MRI analysis tells benign and malignant tumors apart

An automated system for analyzing magnetic resonance imaging (MRI) can differentiate benign from malignant breast tumors with high accuracy. Vishwa S. Parekh and Michael A. Jacobs  from Johns Hopkins University School of Medicine in Baltimore, Maryland, USA, developed an algorithm for extracting textural information from MRI scans that are not visually perceivable to radiologists using machine learning and Radiomic features. Their model combines different MRI parameters to produce so-called radiomic feature maps. The researchers tested their mapping framework on a retrospective cohort of 124 patients, 26 of whom had benign breast lesions and 98 had malignant tumors. They found statistical differences in certain MRI and radiomic metrics. Moreover, they demonstrated quantitative ADC map values and Dynamic contrast pharmacokinetic modeling to characterize the radiomic features. Overall, the method identified a breast lesion as benign or malignant with 93% sensitivity and 85% specificity, suggesting that radiomic feature mapping could aid in diagnosing and characterizing the disease correctly and tailoring therapy accordingly.

## Introduction

Radiomics is an emerging field which deals with the high throughput extraction of quantitative textural features from radiological images.^1–4^ The central hypothesis of radiomics is that by examining the textural features in medical images, it is possible to decode tissue characteristics and pathology. The current radiomic methods extract information about the gray-scale patterns, inter-pixel relationships and shape based properties of the region of interest (ROI).^5–11^ Multiple studies have used radiomic analysis for differentiating between benign and malignant breast tumors,^12–20^ associating radiomic features with histological types of invasive breast cancer^21^ and predicting chemotherapy response in breast cancer patients.^22,23^ In addition, radiomic analysis has been applied to different tissue pathologies such as lung, prostate cancer, and liver cancer, and recently reviewed.^4^ Most of the studies in radiomics are focused on extraction of a single quantitative texture value corresponding to all the voxels within the tumor. As a result, visualization or interpretation of tumor heterogeneity or the correlation between tissue biology of the tumor and the surrounding normal tissue has not been explored. The unique ability of multiparametric magnetic resonance imaging (mpMRI) to better characterize tissue parameters provides us with an opportunity to investigate the correlation between tissue biology and quantitative radiomic metrics. Multiparametric magnetic resonance imaging (MRI) of breast involves acquisition of advanced functional MRI parameters of dynamic contrast enhanced-MRI (DCE-MRI) and diffusion weighted imaging (DWI). In DCE-MRI, a time series acquisition of T1-weighted MRI scans results in time intensity curves corresponding to different tissue types. Moreover, tissue vascularity can be evaluated using pharmacokinetic modeling (PK) of the DCE. Similarly, radiomic analysis of the PK images would produce textural evolution curves which provide information about the underlying vascular “texture” heterogeneity corresponding to different tissue types. Similarly, radiomic analysis applied to the apparent diffusion coefficient (ADC) map obtained from DWI investigates the underlying cellular heterogeneity of the tissue of interest.

To that end, we propose a radiomic feature mapping framework which transforms MRI images into radiomic feature maps for visualization and analysis of textural information present in the images. The radiomic feature maps (RFMs) highlight unique textural information such as contrast, uniformity, heterogeneity, etc. about the radiological images. This information can be correlated with quantitative texture values and quantitative MRI metrics. The motivation behind the development of radiomic feature mapping is to empower the radiologists with the ability to “see” the hidden textural information present in the radiological images and correlate it with tissue biology. Furthermore, we evaluate the textural information of the normal tissue (glandular), benign, and malignant tumors and correlate this textural information with the corresponding vascular and cellular properties of these tissue types. Finally, we analyzed the diagnostic capabilities of RFM features for prediction of clinical diagnosis as benign or malignant using a new multi-view feature embedding and classification model.

## Results

### Experimental summary

**Fig. 1:**
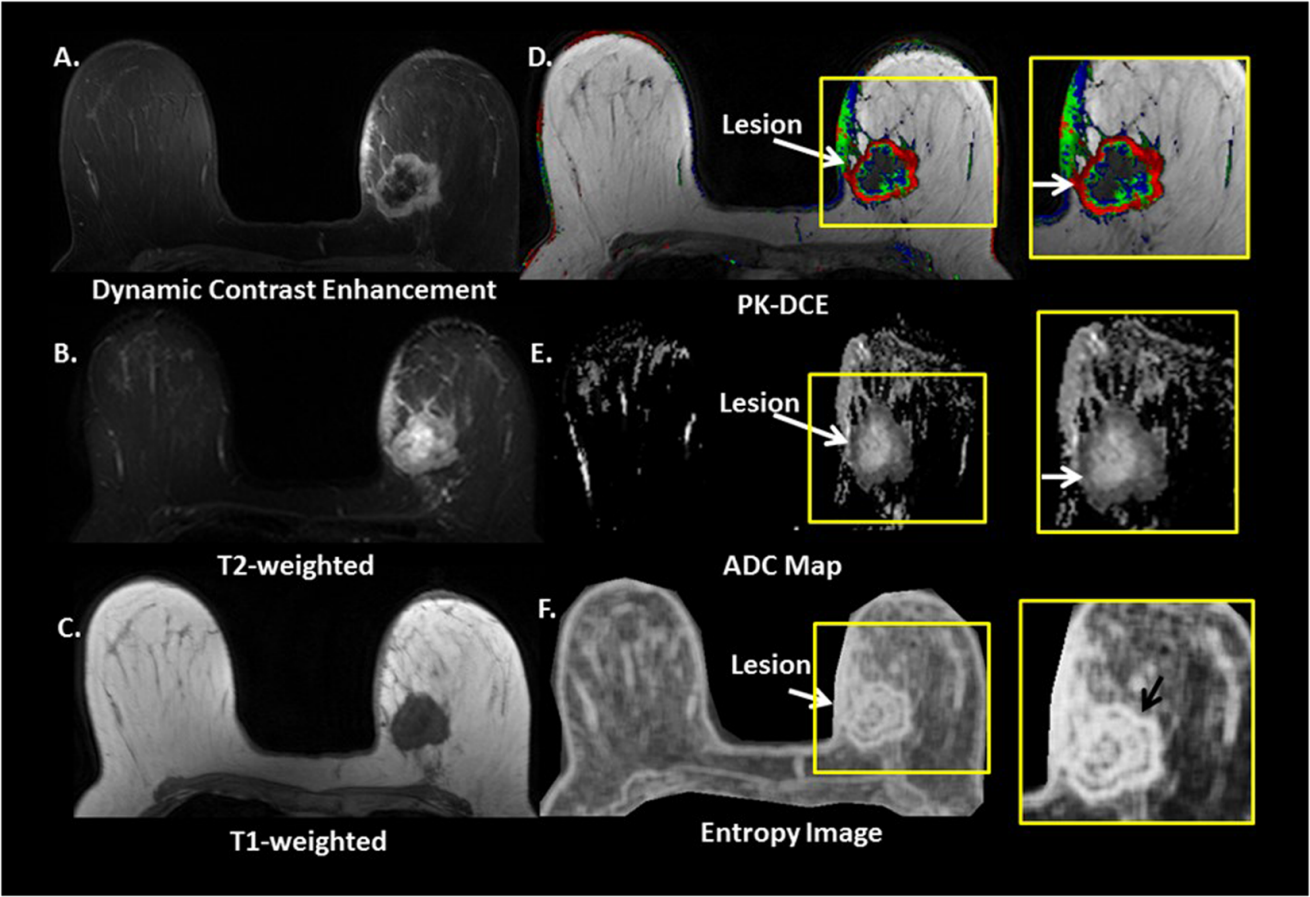
Typical multiparametric breast image of a malignant patient. **a** Dynamic contract enhanced, **b** T2-weighted, **c** T1-weighted, **d** Pharmacokinetic-DCE (PK-DCE) overlay of *K* ^trans^ and EVF, where red indicates high *K* ^trans^ and blue demonstrates low *K* ^trans^ **e** ADC maps, and **f** whole breast entropy feature map

**Fig. 2:**
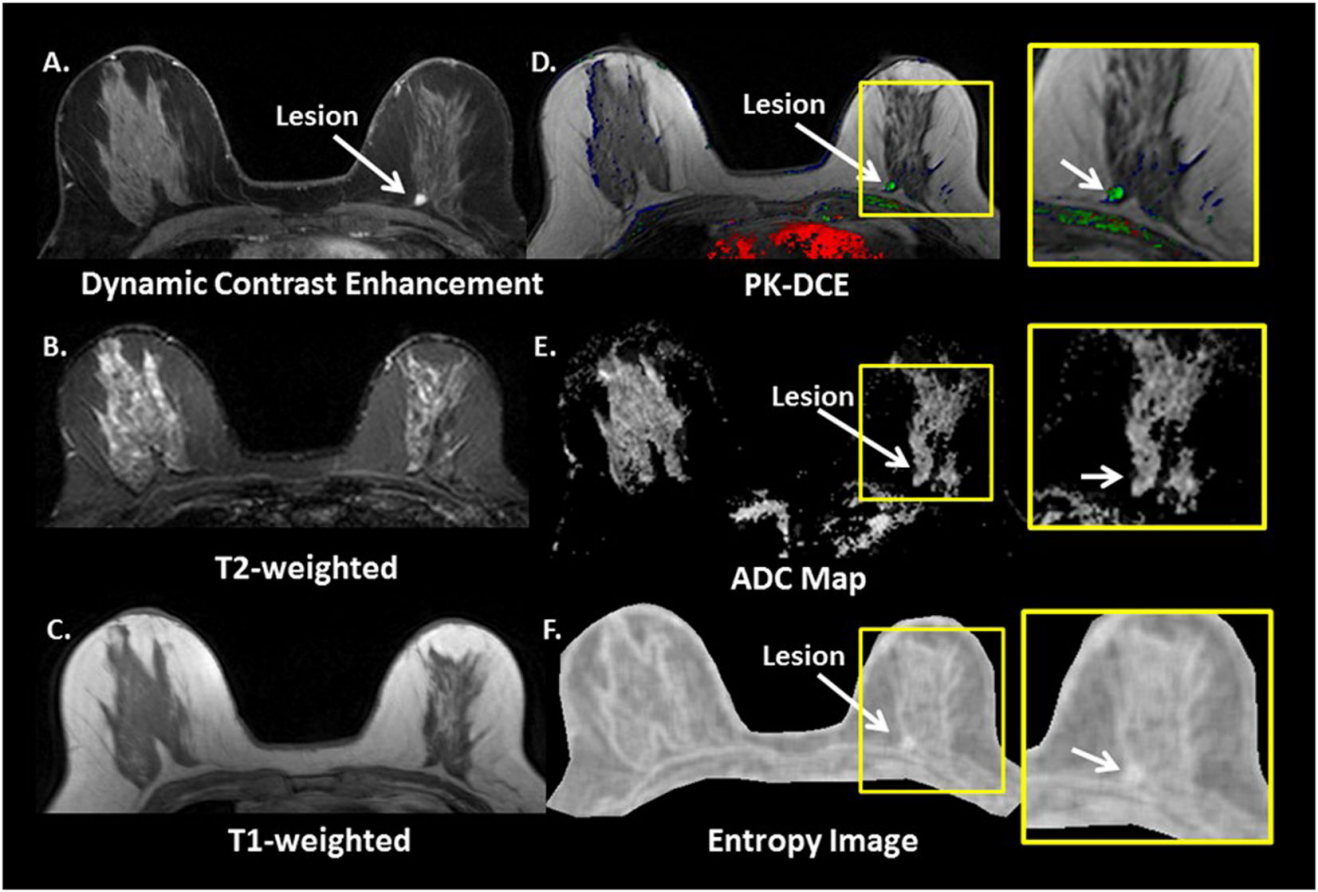
Typical multiparametric breast image of a benign patient. **a** Dynamic contract enhanced, **b** T2-weighted, **c** T1-weighted, **d** Pharmacokinetic-DCE (PK-DCE) overlay of *K* ^trans^ and EVF, where red indicates high *K* ^trans^ and blue demonstrates low *K* ^trans^ **e** ADC maps, and **f** whole breast entropy feature map

**Fig. 3:**
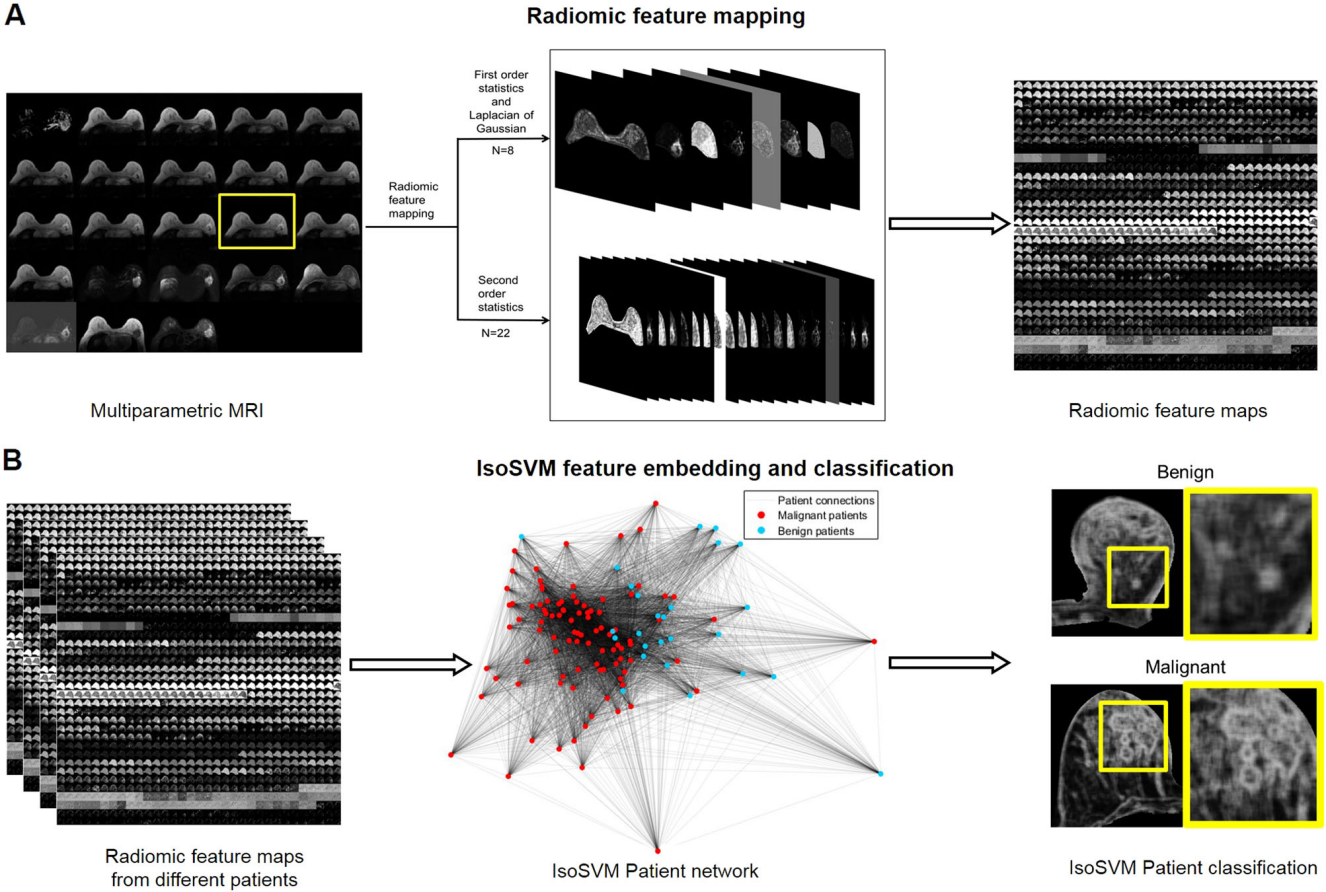
Concept of the radiomic feature mapping framework. **a**. The multiparametric radiological dataset (*N* = 23) is transformed into a high dimensional radiomic feature space (*D* = 690) consisting of radiomic feature maps generated using Laplacian of Gaussian, texture statistical kernels (*n* = 30). **b**. The RFM space is first transformed to patient network using the IsoSVM and then high dimensional radiomic feature map space from each patient is classified as benign or malignant

The radiomic feature maps were computed and analyzed for one hundred and twenty-four women with breast lesions that underwent mpMRI scan. The mean age of the patients was 52 years (range: 24–80 years). Ninety-eight women (79%) had malignant lesions and twenty-six women (21%) had benign lesions. Figures 1 and 2 illustrates typical entropy feature maps corresponding to a benign and a malignant patient. The radiomic features were extracted using our radiomic method that creates whole breast texture images of each feature. The overview of the radiomic feature mapping procedure for classification of a multiparametric radiological dataset as benign or malignant is illustrated in Fig. 3. A total of 690 RFMs were generated for the twenty-three image multiparametric MRI dataset of each patient. Regions of interest were defined on each tissue type using the Eigen filter segmentation method and the MR radiomic feature maps were computed for different breast tissue. Finally, ROI size (area in cm^2^), ADC value and PK-DCE parameters for different regions of interest in each of these patients were obtained. There was no significant difference in the tumor size between benign and malignant patient groups (Benign size: 3.24 ± 2.67 cm^2^, Malignant size: 2.44 ± 0.31 cm^2^, *p* = 0.77).

Table 1 summarizes the entropy values corresponding to the different regions of interest from the DCE-MRI and ADC map. Malignant lesions demonstrated significantly higher entropy on both post contrast DCE-MRI and ADC maps as compared to benign lesions (Benign DCE entropy: 5.72 ± 0.12, Malignant DCE entropy: 6.29 ± 0.06, *p* = 0.0002; Benign ADC entropy: 5.65 ± 0.15, Malignant ADC entropy: 6.20 ± 0.07, *p* = 0.002). There was no significant difference in the glandular tissue entropy values between the two groups (Benign DCE entropy: 6.08 ± 0.10 Malignant DCE entropy: 5.91 ± 0.05, *p* = 0.16; Benign ADC entropy: 6.06 ± 0.32 Malignant ADC entropy: 6.06 ± 0.19, *p* = 1.00).

**Table 1:** Summary of radiomic feature values and quantitative MpMRI metrics

Feature	Glandular tissue	Benign lesion	Malignant lesion	*p* value
Entropy (Post-contrast DCE)	5.95 ± 0.05	5.72 ± 0.12	6.29 ± 0.06	0.0002
Entropy (ADC map)	6.06 ± 0.16	5.65 ± 0.15	6.20 ± 0.07	0.002
ADC map values (mm^2^ × 10^−3^)	2.13 ± 0.03	1.69 ± 0.08	1.26 ± 0.03	0.00001
*K* ^trans^ (min^−1^) values		0.27 ± 0.21	0.69 ± 0.45	0.001
EVF (*V*_*e*_) values		0.27 ± 0.10	0.61 ± 0.31	0.006
Tumor size (cm^2^)		3.24 ± 2.67	2.44 ± 0.31	0.77

*K* ^trans^ = volume transfer constant, *EVF* (*V*_*e*_) extracellular extravascular space

### Analysis of textural evolution curves

**Fig. 4:**
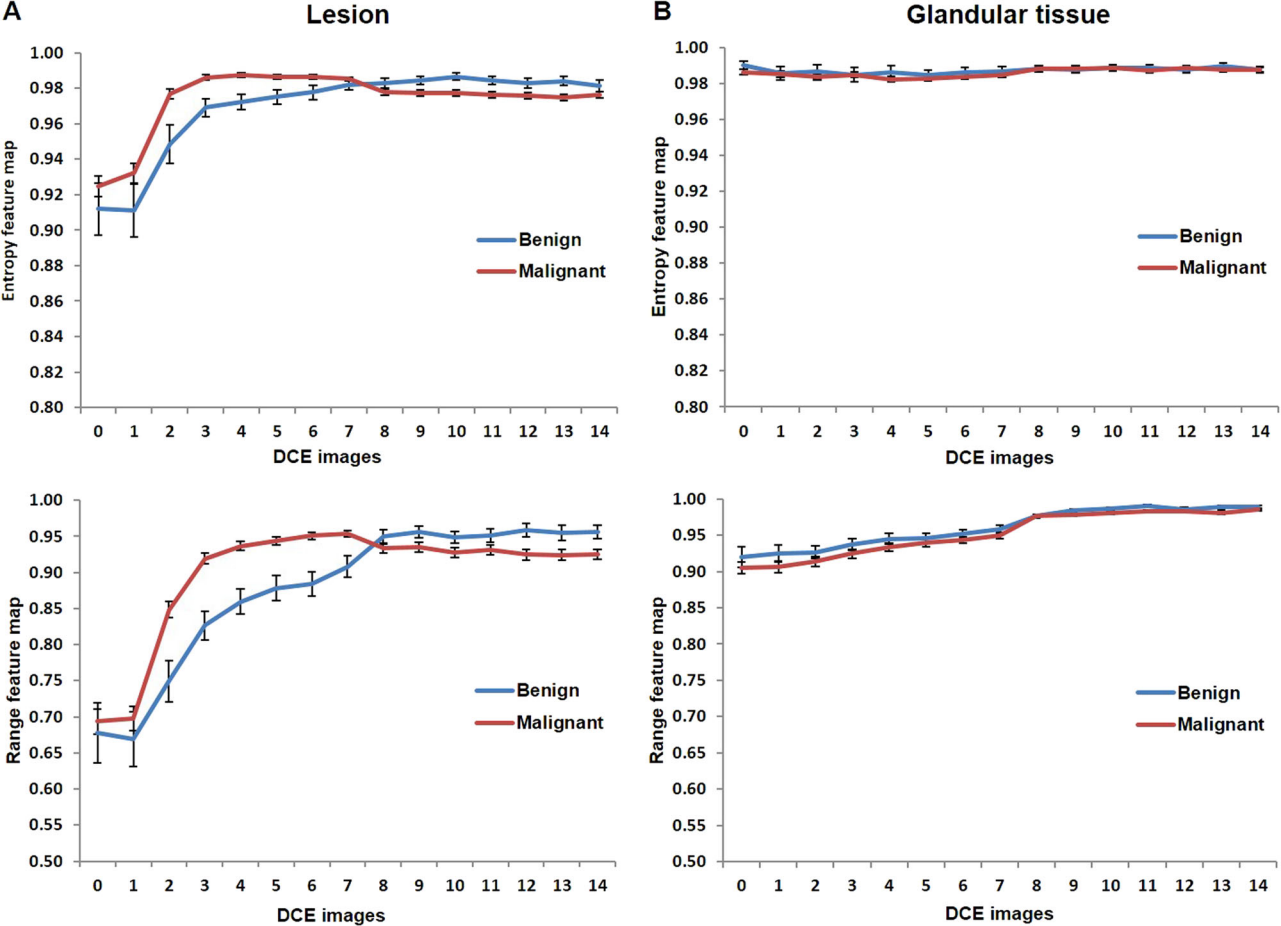
The DCE-MRI entropy evolution curves corresponding to mean value of the entropy feature map and the range feature map. The range feature corresponds to the difference between the maximum and minimum intensity values of all the voxels within the sliding window. The error bars correspond to standard error. (Top) Normalized entropy and (bottom) range feature evolution curves. **a** Lesion graphs of benign (blue) and malignant (red). **b** Contralateral glandular tissue from benign (blue) and malignant patients (red). The shape of the radiomic feature evolution curves were significantly different between the benign and malignant lesions (*p* < 0.05). However, there was no significant difference between the contralateral glandular tissue from benign and malignant patients. Indicative of consistent radiomic features in normal tissue

Figure 4 illustrates the textural evolution curves corresponding to the different radiomic features obtained from DCE MRI. Figure 4a and 4b exhibit the textural evolution curves of the normalized mean values obtained from the entropy feature maps (top) corresponding to tumor and glandular tissue respectively. The error bars in the textural evolution curves represent the standard error for the normalized mean of the entropy values. In Fig. 4b, there is no change in the texture for glandular tissue for both benign and malignant patients. However, the shapes of textural evolution curves were significantly different between benign and malignant lesions illustrating the difference in contrast uptake within benign and malignant lesions. The normalized entropy values during the wash-in phase were significantly (*p* < 0.05) higher for malignant than for benign lesions depicting a rapid textural enhancement for malignant lesions. Similarly, the normalized entropy values during the washout phase were significantly lower for malignant than for benign lesions depicting a rapid textural washout for malignant lesions. Moreover, similar trends were observed in the textural evolution curves obtained from range feature maps as illustrated in Fig. 4 (Bottom). Preliminary analysis of textural evolution curves from entropy feature maps based on the time to peak and the textural wash out slope is shown below.

- Time to peak: The average time to peak for benign lesions (2.21 ± 0.16 mins) was significantly longer (*p* = 0.0003) than for malignant lesions (1.24 ± 0.07 mins).
- Textural wash out slope: The slope of the textural washout curves was also significantly different (*p* = 0.001) between benign (0.001 ± 0.001) and malignant lesions (−0.002 ± 0.0003).

### Analysis of textural evolution metric on DWI

We observed an increase in the first order energy of the lesion tissue from DWI-b0 to b600 with a texture evolution metric that was significantly higher (*p* < 0.001) for malignant (3.09 ± 0.23) than for benign patients (1.84 ± 0.25). Similarly, the contrast in the lesion tissue also increased significantly (*p* = 0.001) for malignant (1.73 ± 0.14) than benign lesions (1.07 ± 0.13). The texture evolution metrics for five different radiomic feature maps portraying different textural characteristics have been summarized in Table 2.

**Table 2:** Summary of the texture evolution metric extracted from different RFMs DWI

	Benign lesion	Malignant lesion	*p* value
Energy	1.84 ± 0.25	3.09 ± 0.23	<0.001
GLCM dissimilarity	0.98 ± 0.06	1.26 ± 0.05	0.001
GLCM contrast	1.07 ± 0.13	1.73 ± 0.14	0.001
GLCM homogeneity	1.10 ± 0.05	0.99 ± 0.02	0.08
First order entropy	0.93 ± 0.02	0.97 ± 0.01	0.12

*GLCM* Gray level co-occurrence matrix

### Multi-view feature embedding and classification

**Fig. 5:**
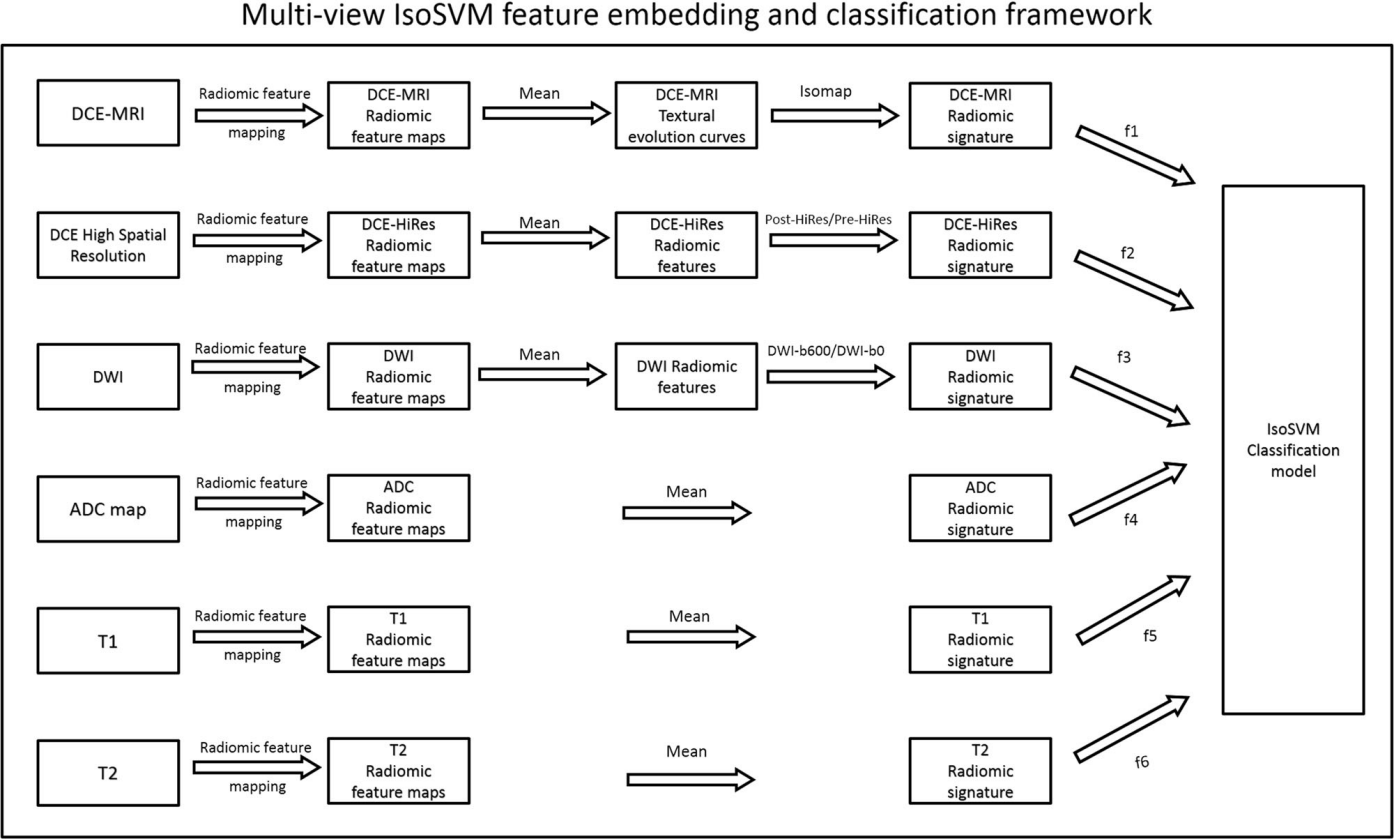
Illustration of the multi-view feature embedding and classification framework. The six MRI datasets are first transformed into radiomic feature map (RFM) space using radiomic feature mapping. The RFMs for DCE-MRI are transformed into textural evolution curves, which are subsequently reduced to one dimensional embedding using the Isomap algorithm. The vector of one dimensional embedding corresponding to each RFM forms the 30 dimensional DCE-MRI radiomic signature. The RFMs for DCE High spatial resolution MRI and DWI are transformed into their respective radiomic signatures based on the textural evolution metric. The remaining datasets of ADC map, T1WI, and T2WI are directly transformed into radiomic signatures by calculating the mean of the RFMs. Finally, subsets of features (f1, f2,…,f6) from each RFM signature form a unified RFM signature used to train the IsoSVM Classification model

The multi-view feature embedding and classification framework was set up as illustrated in Fig. 5. The optimal set of hyperparameters for the multi-view classification framework, obtained using leave one out cross validation based grid search, were f1 = 18, f2 = 0 f3 = 6, f4 = 8, f5 = 0, f6 = 0, neighborhood parameter *k* = 45, dimensionality *d* = 10 and misclassification penalty ratio = 2.5:1. The parameter space for each of the input parameters were set as follows:

- The subset of features, f_i_ selected from each MRI dataset were iteratively selected based on the area under the ROC curve computed using univariate logistic regression.
- The neighborhood parameter was varied from 5 through 120 in steps of 5.
- The dimensionality of the transformed feature space was varied between one and ten.
- The misclassification penalty ratio between benign and malignant classes was selected from the set {2:1, 2.5:1, 3:1, 3.5:1, 4:1}.

**Fig. 6:**
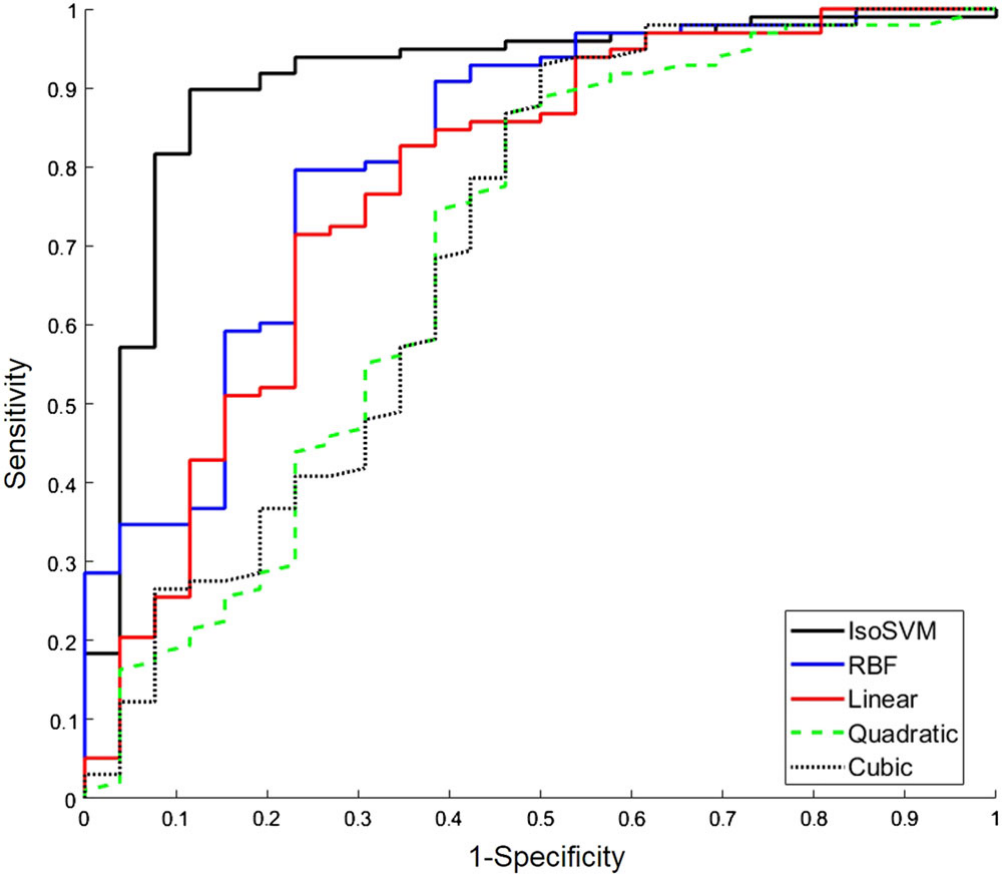
The receiver operating characteristic curves corresponding to the IsoSVM classification (black), radial basis function (RBF) kernel SVM (blue), linear kernel SVM (red), quadratic kernel SVM (dashed green) and the cubic kernel SVM kernel (dotted black) evaluated using leave one out cross validation. The area under the ROC were obtained at 0.91, 0.82, 0.78, 0.65, and 0.71 for IsoSVM, RBF, linear, quadratic, and cubic kernel SVMs, respectively

The multi-view feature embedding and classification model trained using leave-one-out cross validation resulted in sensitivity and specificity of 93 and 85%, respectively, with an AUC of 0.91 in classifying benign from malignant lesions. The ROC curves for the IsoSVM classification model and other kernels are shown in Fig. 6. The search space for the misclassification penalty parameter for the SVM kernels was increased to all the ratios in the set {2:1, 2.5:1, 3:1,3.51, 4:1, 4.5:1,5:1,5.5:1,6:1} The resultant sensitivity, specificity and AUC from all the SVM classifiers are shown in Table 3. The multi-view feature transformation and classification framework was further tested using ten-fold cross validation performed across 100 trials. The optimal set of hyperparameters obtained with ten-fold cross validation concurred with the previously obtained optimal set of hyperparameters using leave one out cross validation. The average sensitivity and specificity achieved from ten-fold cross validation experiment were 91 and 82%, respectively, with an AUC of 0.87. The result from ten-fold cross validation ascertains the stability of the unified RFM signature, as well as the IsoSVM classifier. For comparison, the classification of benign from malignant using tumor size alone produced an AUC of 0.77 which was significantly lower than the AUC of 0.91 obtained from the unified RFM signature using the IsoSVM classifier.

**Table 3:** Summary of sensitivity, specificity and AUC for the IsoSVM classifier and various SVM kernels

Classifier	Input parameters	Sensitivity	Specificity	AUC
IsoSVM	*k *= 45; *d* = 10; PR = 2.5:1	0.93	0.85	0.91
Radial basis function SVM	Sigma = 19; PR = 5:1	0.80	0.77	0.82
Linear SVM	PR = 3.5:1	0.85	0.62	0.78
Quadratic SVM	PR = 5.5:1	0.85	0.54	0.65
Cubic SVM	PR = 5:1	0.93	0.50	0.71

*AUC* area under the curve, *IsoSVM* hybrid isomap and support vector machine, *PR* the misclassification penalty ratio between benign and malignant classes

## Discussion

We have demonstrated that our radiomic feature maps for visualization and evaluation of radiological texture in radiological images produced excellent features that were correlated to breast tissue biology and compared with quantitative metrics of radiological parameters. Malignant lesions demonstrated increased entropy compared to benign lesions for both ADC maps and DCE MRI. In contrast, glandular tissue entropy was similar across all subjects. Furthermore, the radiomic feature maps (RFMs) demonstrated excellent sensitivity and specificity in classifying benign from malignant lesions. Moreover, this study relates the quantitative metrics of ADC maps and PK-DCE to radiomics values for characterization of breast lesions and normal tissue.

Radiomic features, such as, entropy have been shown to classify between benign and malignant tumors in addition to predicting patient survival and treatment response in previous studies as reviewed in ref. 4. However, our work explored the whole image visualization and interpretation of these quantitative radiomic values employing RFMs. Indeed, the entropy feature maps exhibit higher entropy and intra-tumor heterogeneity for malignant tumors compared to benign tumors. The RFMs would provide the radiologists with a tool for visual interpretation of the radiomic feature values. Furthermore, radiomic feature maps provide a visualization of intra-tumor heterogeneity as opposed to a single quantitative value provided by quantitative radiomic analysis. In addition, radiomic feature maps produce voxel-wise radiomic values improving the quantitative measure. In contrast, single quantitative value corresponding to the whole tumor region may not define the entire tumor. This study investigated the relationship between RFMs and underlying tissue biology derived from quantitative radiological images. Preliminary analysis of RFMs corresponding to DCE-MRI suggest that time evolution of RFMs is indicative of heterogeneity in the vasculature of the tissue  microenvironment. We observed that the textural evolution curves obtained from mean value of the radiomic feature maps had significantly different curve characteristics for benign and malignant tumors. Furthermore, the glandular tissue corresponding to benign and malignant patients demonstrated no shape difference, indicating there is no textural evolution difference with contrast uptake within glandular tissue. The radiomic features provided new metrics for comparison of the different tissue types. Moreover, the vascular parameters of *K* ^trans^ and EVF have been shown to be different between benign and malignant tumors. In concordance, the radiomic values also demonstrated significant differences between tissue types. In the previous studies, the ADC value and PK-DCE for a given region of interest has been established as an excellent biomarker in classification between benign and malignant breast tumors.^24–31^ Here, we establish radiomic entropy (and others) of the ADC map and DCE-MRI within the tumor ROI as a biomarker for correlation with cellular and vascular heterogeneity. The ADC and DCE entropy was significantly different between benign and malignant tumors. Furthermore, the entropy ADC feature map provides more insight into the cellular distribution within the tumor, whereas, the DCE radiomic metric provides information about the vascularity texture of the tissue. Additionally, a metric for quantification of tissue heterogeneity evolution with increasing *b* value was developed and analyzed. A subset of the texture evolution metrics for DWI were significantly different between benign and malignant lesions indicating a potential biomarker in the texture evolution metric. In addition, the glandular ADC and radiomics values were similar across all subjects. These findings lay the groundwork in radiomic metrics to describe normal vs abnormal tissue, which is needed for increased use of radiomics in clinical applications.

The training efficacy of most machine learning algorithms depend on the balance between the number of instances corresponding to each class. Typically, benign breast tumors are more frequently observed in clinical setting as compared to malignant tumors. However, in research setting, MRI for malignant breast tumors are more frequently obtained than for benign breast tumors producing a class imbalance that may result in performance bias of the trained classifier towards one class. Class imbalance is a frequent occurrence in health care machine learning applications. Our work approached the problem of class imbalance by assigning different misclassification penalty to each class type. Our results indicate that setting an appropriate misclassification penalty significantly improves the classification accuracy.

Our work has certain limitations. First, the radiomic feature map creation and classification were performed on retrospective data and no separate validation data was used. Second, this study evaluated radiomic feature maps corresponding to only first and second order statistical features. Other statistical radiomic methods such gray level run length matrix features,^8^ Neighborhood gray tone difference matrix feature^10^ have not been evaluated in this study. Third, RFMs provide voxel wise heterogeneity information of the whole tissue of interest. However, the feature used in the texture evolution curves and classification model was the mean derived from the RFMs. In the future studies, the goal would be to evaluate each voxel of interest and produce voxel-wise classification.

In summary, RFMs present a new powerful tool for analysis of textural information present within anatomical and quantitative radiological images and may provide a new perspective into the biological information that radiomics is capable of providing and the potential it holds in future diagnostic applications.

## Conclusion

This work presents radiomic feature maps (RFMs) for visualization of textural information present in anatomical and quantitative radiological images. The correlation between the quantitative radiological biomarkers (ADC and PK-DC) with radiomic values provided by RFMs was established in this paper. Our results suggest that the textural evolution curves obtained from DCE-MRI RFMs were significantly different for benign and malignant tumors establishing a correlation with vascular heterogeneity. Similarly, the cellular heterogeneity, evaluated using entropy of the ADC map along with textural evolution metric on DWI was significantly different between malignant and benign tumors. Finally, the IsoSVM feature selection and classification model achieved excellent sensitivity and specificity in comparison to state of the art classification methods despite highly imbalanced data.

## Materials and methods

### Clinical data

The radiomic feature mapping framework was tested on a multiparametric MRI dataset obtained from a retrospective cohort of 124 patients (mean age = 52, range = 24–80) to classify between malignant and benign lesions. Out of the 124 patients, 98 had malignant lesions while 26 patients had benign lesions. Patients were selected for the study based on the potential for malignant breast lesions with a BIRADS score of 3 or greater from 2008–2010. This retrospective study was approved by the IRB at our facility and conforms to HIPAA requirements.

### Multiparametric MRI imaging protocol

All patients were scanned on a 3-T clinical MRI system using a bilateral dedicated 4 channel phase-array breast coil in the prone position. MRI sequences acquired included ultrafast spoiled gradient echo (T1-TFE) T1-weighted images (TR/TE: 5.37/2.3 ms; Slice thickness (ST) = 3 mm; Field of view (FOV): 35 × 35 cm; Flip angle (FA) = 12^0^) and fat suppressed (FS) T2-weighted spin echo images (TR/TE: 6122/70 ms; ST = 4 mm; FOV:35 × 35 cm; FA = 90^0^). The DCE-MRI was obtained using FS and non-FS, three-dimensional, FSPGR T_1_-weighted (TR/TE = 4.2/2.1 ms; FOV:35 × 35 cm; ST = 5 mm) sequences. One non-FS pre-contrast and fourteen post-contrast images (15 secs per acquisition) for PK analysis were obtained after intravenous administration via a power injector of a GdDTPA contrast agent (0.2 mL/kg(0.1 mmol/kg)).^32,33^ Two minutes of T1 fat-suppressed high temporal resolution (15 sec per acquisition) imaging was obtained to capture the wash-in phase of contrast enhancement, followed by a high spatial resolution scan for 2 min. Diffusion weighted imaging was obtained using an FS spin echo Echo Planar Imaging sequence (TR/TE = 5000/90 ms, SENSE = 2, ST = 3–4 mm, *b* = 0–600 s/mm^2^) on three planes.

### Quantitative MR image analysis

ADC maps were constructed from DWI using a monoexponential equation. DCE Subtraction images were constructed by subtracting pre-contrast from the post-contrast high spatial resolution DCE images.

### Dynamic MRI with Pharmacokinetic (PK) Contrast Enhancement

The vascularity of breast tissue was obtained using different semi-quantitative and quantitative metrics.^34,35^ The semi-quantitative metrics use the temporal evolution of the time series curves from the DCE MR images and are scaled into three categories relating to the potential characterization of the tissue and other known metrics.^32,36,37^

The PK-DCE quantitative metrics derived were volume transfer constant (*K* ^trans^ (min^−1^)), the fractional volume of the extracellular extravascular space (EVF (*V*_*e*_)), and the transfer rate constant (*k*_*ep*_ (min^−1^)) using commercial software DynaCad (InVivo, Gainesville, Florida).^32,33^ For both benign and malignant patients, glandular and lesion tissue, the mean values and standard deviations of the transfer constant (*K* ^trans^) extra-vascular volume fraction-EVF (*V*_e_) were recorded.

### Registration

The mpMRI images were co-registered using the algorithm developed in ref. 38. The registration algorithm reduces information loss during rescaling and reslicing of the MRI volumes using a 3D wavelet transformation. The pre-contrast DCE image was used as the reference registration image for all other images.

### Image segmentation

Normal glandular tissue, benign and malignant tumors were segmented from the mpMRI dataset using the Eigenfilter algorithm.^39^ Eigenfilter is a well-established semi-automatic segmentation algorithm based the Gram-Schmidt orthonormalization algorithm using tissue signatures to define different tissue types.^40–42^

### Radiomic feature mapping

The radiomic feature mapping framework transforms each MRI image into a multidimensional radiomic feature map space (RFMS), defined as RFMS={*RFM*_1_, *RFM*_2_,…,*RFM*_*N*_ } ∈ *R* ^*D*^,where RFM_i_ represents the ith radiomic feature map (RFM), *D* represents the number of voxels in the MRI image and N represents the number of RFMs generated. The RFM framework algorithm that transforms radiological data into the RFMS is defined below(Fig. 3). First, a set *S* of *N* radiomic filters from different radiomic features is generated. The size of the radiomic filters or the neighborhood scaling parameter, *W* is determined by the user depending on the spatial resolution of the input MRI image. Second, the quantization of the MRI image intensities to *G* levels for radiomic first and second order statistics require the MRI image intensities to be quantized. The value of *G* is determined by the user based on the range of intensities, as well as the number of bits required to represent voxel intensity in the input image. In the final step, the MRI image is convolved with each of the *N* radiomic filters in the set, *S* to produce *N* radiomic feature maps. As a result, every voxel in the original MRI image has a corresponding radiomic feature value in each RFM. The mean of the radiomic values were calculated from different regions of interest (ROI) in each RFM as features for classification and further analysis. Consequently, every RFM feature from every patient corresponds to the average value taken from sliding same sized image window (*W* x *W*) across the whole ROI ensuring there is no mathematical dependence between the computed RFM features and size of the ROI.

The input parameters for this study were *G* = 256, *W* = 9, and *N *= 30. Out of 30 RFMs, 7 were generated using first order statistics, 22 were generated using second order statistics and one was generated using Laplacian of Gaussian filter.

### Comparison between radiomic feature maps, quantitative radiomic metrics, and MRI metrics

Single quantitative radiomic values corresponding to benign lesion, malignant lesion, and glandular tissue were computed. The quantitative radiomic values corresponding to each ROI were compared with the functional metrics from the DWI and PK-DCE MRI images for their diagnostic ability to classify between benign and malignant lesions using a paired *t*-test and univariate logistic regression.

### Textural evolution curves

The PK-DCE MRI RFMs provided information on the vascular heterogeneity within lesion tissue. The RFM textural evolution curves capture the time evolution of tissue heterogeneity as a function of contrast uptake using the time series derived from DCE images. The mean radiomic feature value across all the voxels within a region of interest in the RFMs was used to construct textural evolution curves for each tissue type. In order to compare the textural evolution curves across different ROIs, normalization of the radiomic feature values were applied.

Analysis of textural evolution curves from entropy feature maps were done using the time to peak and the textural wash out slope from the DCE RFMs. The time to peak is defined as the time it took for the textural evolution curves to reach its maximum value. The textural wash out slope is as the textural wash out of the textural evolution curves within the lesion tissue. This was computed as the slope of the line connecting the peak texture enhancement in the first 2 min to the last time point including all the intermediate time points.

### Textural evolution metric for DWI

The textural evolution metric (TEM) was developed to investigate the change in tissue heterogeneity of different tissue types. We defined a textural evolution metric (TEM) for DWI as follows:

$$TE{M_{DWI}} = \frac{{RF{M_{b  >0}}}}{{RF{M_{b0}}}}$$

Similarly, a textural evolution metric (TEM) was defined for the high spatial resolution DCE-MRI dataset (HR-DCE-MRI) as shown here:

$$TE{M_{HR - DCE - MRI}} = \frac{{Pos{t_{HR - DCE - MRI}}}}{{Pr{e_{HR - DCE - MRI}}}}$$

### Statistical analysis

We computed summary statistics (mean and standard error of the mean) for the radiomic metrics and functional metrics from mpMRI. An unpaired t-test was performed to compare the RFMs for the benign and malignant patient datasets. Statistical significance was set at *p* ≤ 0.05. Univariate logistic regression analysis was used to find associations between the RFMs and the final diagnosis. Receiver operating characteristic (ROC) curve analysis was performed to assess the diagnostic performance of each RFM in characterizing benign vs. malignant lesions.

### Multi-view IsoSVM framework for feature embedding and classification

The radiomic feature maps were computed from a mpMRI dataset resulting in a high dimensional feature space. Furthermore, the radiomic feature maps corresponding to different imaging sequences highlight different functional textural properties of the tissue of interest. Consequently, we developed a multiview feature embedding and classification framework termed IsoSVM by modifying and combining the Isomap^43^ and support vector machine (SVM) algorithms.^44^ The overview of the IsoSVM framework is shown in Fig. 5.

### Computation of radiomic signatures

The high dimensional mpMRI radiomic feature space was first analyzed to compute six different radiomics signatures as follows:

- PK-DCE MRI radiomic signature: The textural evolution curves corresponding to the radiomic feature maps were transformed into a radiomic signature using the Isomap algorithm.^43^ For the PK-DCE RFM dataset, the 15 dimensional textural curves were transformed into a single dimensional representation of the textural evolution curve characteristic. The correlation coefficient between the textural evolution curves of different patients was used as the distance metric to compute the geodesic distances for the low dimensional embedding.
- DWI and DCE-MRI High spatial resolution radiomic signatures: The vector of the textural evolution metrics for the radiomic feature maps was used as the radiomic signature for both the datasets.
- ADC map, T1WI, and T2WI radiomic signatures: Each one was a single image, making the vector of the mean of the radiomic feature maps, their radiomic signature.

### Feature selection

The set of radiomic signatures from the MRI datasets resulted in a 180-dimensional radiomic feature space. The 180-dimensional radiomic feature space was then transformed and modeled into an IsoSVM classification model as detailed below.

- The feature set from each of the radiomic signatures was sorted from largest to smallest based on the area under the receiver operating characteristic curve obtained using univariate logistic regression.
- A subset of top features (f1, f2, …, f6) from each of the radiomic signatures were selected to create a unified radiomic signature, *g *= U_*fi*_.
- The unified radiomic signature, *g* was then transformed into a linearly separable, low dimensional feature space, h using the Isomap algorithm. The feature transformation was executed using Isomap because the Isomap algorithm is not prone to overfitting because of its unsupervised nature and at the same time, accounts for the dependencies between different RFMs.

### Classification

- The support vector machine algorithm trains a classification model to classify between benign and malignant patients on the transformed feature space, *h*. Because SVM is a linear binary classification algorithm that attempts to create a hyperplane that best separates the different groups, the application of Isomap algorithm prior to SVM reduces the non-linearity in the data by transforming the feature space, *g* to *h*. The steps c and d combine to form the hybrid IsoSVM classification model. Mathematically, the hybrid IsoSVM classifier is represented using the following equation:
  $$f\left( x \right) = \mathop {\sum }\limits_{i = 1}^N {\alpha _i}{y_i} < \phi \left( {{x_i}} \right),\phi \left( x \right) > + b$$
  where *φ()* is the Isomap transformation function that maps the unified radiomic signature, g into a linearly separable space, *h*, *N* is the number of patients in the training set, *α*_*i*_ are the Lagrange multipliers, *x*_*i*_ represents the radiomic signatures of training set patients and *x* represents the radiomic signature of the test patient. *y*_i_ represents the classes where *x*_i_ resides.
- For comparison, we tested five different SVM kernels on the unified radiomic signature, *g* including the hybrid IsoSVM kernel to classify the benign and malignant patients to determine the optimal kernel.
- Finally, due to class imbalance between the number of benign and malignant patients, the ratio between penalty for misclassification of different patient data sets was varied to identify the optimal penalty ratio and shown in the supplementary data. ^45^

The complete set of input parameters (input feature space: f1, f2, …,f6; Isomap neighborhood parameter, *k*; Isomap dimensionality, *d*; and misclassification penalty ratio) were estimated using leave-one-out and *k* fold cross validation (*k* = 10).

### *K*-fold cross validation

The set of benign and malignant patients were first separately divided into ten randomly sampled subsets due to imbalance in the number of patients in each patient group. Next, the ten subsets from both categories were combined to form ten patient subsets. As a result, the ratio between the number of benign and malignant patients was maintained similar to the original patient cohort in the patient subsets. The ten-fold cross validation procedure was performed on these ten subsets. The complete procedure of generating ten subsets and performing ten-fold cross validation was repeated 100 times to avoid any bias that may occur due to specific partitioning of the data.

### Code availability

Our software will be freely available to academic users after issue of pending patents and a materials research agreement is obtained from the university. Due to University regulations, any patent pending software is not available until a patent is issued.

### Data availability

All relevant clinical data are available upon request with adherence to HIPPA laws and the institutions IRB policies.

## Electronic supplementary material

Supplementary Materials
