# Supplementary material for: Integrated radiomic framework for breast cancer and tumor biology using advanced machine learning and multiparametric MRI
Source: NPJ Breast Cancer. 2017 Nov 14;3:43. doi: 10.1038/s41523-017-0045-3 (PMC5686135; doi:10.1038/s41523-017-0045-3)
Supplement: Supplementary file 1 — Supplementary Materials [file 41523_2017_45_MOESM1_ESM.docx]

**Supplementary methods**

**Cost sensitive learning for IsoSVM**

A majority of classifiers including SVM^1^ are trained under the assumption that the training sets are well balanced. The prior probability of classification of different classes is calculated empirically based on the frequency of occurrence of each class in the training set. However, if the data distribution in the training set is highly skewed towards one class, the classifier tends to be biased towards the more dominant class to increase the classification accuracy. For our data, the prior probabilities for the malignant and benign classes are calculated empirically at 0.79 and 0.21 respectively. Thus, the imbalance in the prior probability would cause the classifier to be highly sensitive but not very specific i.e. the classifier will try to maximize the accuracy by predicting every example as malignant.

To overcome the problem of class imbalance, we implemented cost sensitive learning^2^ where the prior probabilities (p_b_ for benign and p_m_ for malignant) are modified based on an additional user input: misclassification penalty. Mathematically, cost sensitive learning is implemented as follows:

Let the misclassification penalty for benign and malignant classes be initialized as C_b_ and C_m_ respectively. The new prior probabilities $(p_{b}^{new}$ & $p_{m}^{new})$ are then computed using the following equations:

$p_{b}^{*}=p_{b}\times C_{b}$ (1)

$p_{m}^{*}=p_{m}\times C_{m}$ (2)

$p_{b}^{new}=\frac{p_{b}^{*}}{p_{b}^{*}+p_{m}^{*}}$ (3)

$p_{m}^{new}=\frac{p_{m}^{*}}{p_{b}^{*}+p_{m}^{*}}$ (4)

Equations 3 and 4 are based on the law of total probability.

As an example, if the misclassification penalty ratio is set to 3.5:1 (benign:malignant), then the prior probabilities will be updated from 0.79 to 0.52 for malignant class and 0.21 to 0.48 for benign class. This would make the priors more balanced relative to the “skewed coin toss” owing to the initial values of prior probabilities.

**Subsampling analysis**

We divided the malignant patients into four groups based on the tumor size as shown in table 1. We performed two experiments on the subsampled patient groups to study the effect of tumor size and class imbalance on the IsoSVM classification results.

Experiment 1: We trained and evaluated four IsoSVM classifiers on each of the four malignant patient groups using leave one out cross validation. This experiment was performed to study the effect of tumor size on IsoSVM classification results.

Experiment 2: We trained and evaluated four IsoSVM classifiers on four combinations of malignant patient groups using leave one out cross validation. Here, the first malignant patient dataset composed of group 1, second composed of groups 1 and 2, third composed of groups 1, 2 and 3 and the final dataset composed of all patients. This experiment was performed to study the combined effect of tumor size and class imbalance on the IsoSVM classification results.

**Supplementary Results**

The IsoSVM model achieved excellent sensitivity, specificity and area under the curve (AUC) for all the groups demonstrating the diagnostic power of the radiomic feature maps. All the four malignant patient groups demonstrated high sensitivity and specificity as shown in table 2 suggesting that there is no dependence between the RFM features and tumor size. Furthermore, the class imbalance between benign and malignant patients was also successfully overcome using different misclassification penalty ratios as shown in table 3.

**Supplementary tables.**

Table 1. Summary of the number of patients and average tumor size for the four malignant patient groups created for subsampling analysis.

| **Dataset** | **Number of malignant patients** | **Average tumor size (cm^2^)** |
| --- | --- | --- |
| **Group1** | 26 | 0.33±0.03 |
| **Group 2** | 26 | 0.99±0.04 |
| **Group 3** | 26 | 2.29±0.14 |
| **Group 4** | 20 | 7.27±0.81 |

Table 2. Summary of sensitivity, specificity and AUC for the IsoSVM classifier for the four malignant patient groups. The misclassification penalty ratio between benign and malignant classes is denoted with PR. The Isomap neighborhood parameter and the dimensionality of the transformed low dimensional embedding are indicated by k and d respectively.

| **Malignant Dataset** | **Benign patients** | **Input parameters** | **Sensitivity** | **Specificity** | **AUC** |
| --- | --- | --- | --- | --- | --- |
| **Group1** | All | k=5; d=8; PR=1.5:1 | 0.88 | 0.88 | 0.90 |
| **Groups 2** | All | k=30; d=3; PR=2:1 | 0.88 | 0.85 | 0.86 |
| **Groups 3** | All | k=15; d=10; PR=1.5:1 | 0.85 | 0.85 | 0.89 |
| **Group 4** | All | k=30; d=10; PR=1:1 | 0.85 | 0.81 | 0.88 |

Table 3. Summary of sensitivity, specificity and AUC for the IsoSVM classifier for the four malignant patient datasets created by incrementally combining the four malignant patient groups. The misclassification penalty ratio between benign and malignant classes is denoted with PR. The Isomap neighborhood parameter and the dimensionality of the transformed low dimensional embedding are indicated by k and d respectively.

| **Dataset** | **Benign patients** | **Input parameters** | **Sensitivity** | **Specificity** | **AUC** |
| --- | --- | --- | --- | --- | --- |
| **Group1** | All | k=5; d=8; PR=1.5:1 | 0.88 | 0.88 | 0.90 |
| **Groups 1 and 2** | All | k=25; d=10; PR=1.5:1 | 0.88 | 0.81 | 0.90 |
| **Groups 1, 2 and 3** | All | k=35; d=10; PR=2.5:1 | 0.87 | 0.81 | 0.89 |
| **All patients** | All | k=45; d=10; PR=2.5:1 | 0.93 | 0.85 | 0.92 |

References

1 Cortes, C. & Vapnik, V. Support-vector networks. *Machine learning* **20**, 273-297 (1995).

2 Elkan, C. The foundations of cost-sensitive learning. *International joint conference on artificial intelligence* **17**, 973-978 (2001).
